# Cognitive Enhancement through Improved Central Artery Stiffness in Postmenopausal Women: Potential Benefit of High-Intensity Aerobic Exercise

**DOI:** 10.18502/ijph.v49i8.3905

**Published:** 2020-08

**Authors:** Moon-Hyon HWANG

**Affiliations:** 1. Division of Health & Kinesiology, Incheon National University, Incheon, South Korea; 2. Sport Science Institute, Incheon National University, Incheon, South Korea

## Dear Editor-in-Chief

With advancing age, the incidence rate of Alzheimer’s disease and dementia gradually increases, and mortality from either Alzheimer’s disease or dementia has increased in recent decades (1). Recently, one third of adults older than 65 yr of age die from either Alzheimer’s disease or dementia(1). Mild cognitive impairment (MCI) is a precursor to both Alzheimer’s disease and dementia. In general, MCI occurs more frequently in women compared to men, and takes place more frequently in postmenopausal women than premenopausal women (2). Cognitive impairment is associated with cardiovascular dysfunction. The MCI risk of patients with cardiovascular disease is 77% higher than for persons without cardiovascular disease (3).

Postmenopausal women experience a dramatic decrease in circulating estrogen level. Due to the cardio-protective effect of estrogen, premenopausal women are exposed to a lower risk of cardiovascular disease compared to postmenopausal women. Elastic conduit arteries including the aorta are more vulnerable to either internal or external stimulation than smaller muscular vasculatures because of their anatomical characteristics. Thus, central artery stiffness measures such as pulse wave analysis (augmentation index) and aortic pulse wave velocity are used as validated surrogate markers to predict future cardiovascular morbidity and mortality. Elastic central arteries in postmenopausal women older than 60 yr of age are in particular stiffer than pre- or postmenopausal women under 60 yr of age (4). In a recent review, the linkage of vascular dysfunction to cognitive impairment was suggested, based on de la Torre’s hypothetical model (3). Briefly, vascular dysfunction induces disrupted hemodynamics, which influences cognitive function via negative alterations in cerebrovascular perfusion. Considering the above-mentioned hypothesis, it seems to be plausible that augmented central artery stiffness in postmenopausal women plays a mediating role in cognitive impairment via disordered hemodynamics and perfusion between the heart and brain regions.

Regular physical activity including aerobic and resistance exercise is recommended for postmenopausal women to improve not only musculoskeletal and cardiovascular function, but also cognitive function. Three months of combined circuit exercise reduce arterial stiffness and other cardiovascular disease risks in hypertensive postmenopausal women(5). Furthermore, older adults who exercise regularly more than three times a week have lower risk for dementia (3). Exercise volume, mode, and intensity may influence cognitive function in postmenopausal women. The volume of physical activity in older adults is important because it is inversely related to the cognitive impairment and dementia risk for the following decade (3). Compared to resistance exercise, aerobic exercise seems more beneficial for cognitive enhancement in that steady-state aerobic physical activity increases brain blood flow through augmented oxygen supply from the heart. Exercise intensity plays a critical role in regulating brain blood flow. Moderate to high-intensity aerobic exercise causes a greater increase in blood circulation in both cardiovascular and cerebrovascular regions compared to low-intensity aerobic exercise (6). Exercise-induced augmentation of blood flow in the brain is closely associated with both cognitive function and the increased protein expression of neurotrophic factors such as brain-derived neurotrophic factor (BDNF), insulin-like growth factor-1, and vascular endothelial growth factor (VEGF) in the brain and peripheral tissues (6).

Compared to continuous exercise, high-intensity interval exercise can be more beneficial in enhancing cognitive function via not only decreased central artery stiffness but also facilitated neurotrophic factors production and increased neurotrophic factor bioavailability in postmenopausal women. The hypoxia state in high-intensity aerobic exercise increases VEGF production. In particular, intermittent hypoxia for high-intensity interval exercise facilitates the generation of BDNF, the most important neuroplasticity marker, in the cerebrovascular endothelium. Furthermore, lactate, a byproduct of high-intensity exercise, can be used to facilitate BDNF and VEGF generation in peripheral tissues and is an additional energy resource for brain neuron activity. Both the BDNF and VEGF produced in peripheral tissues can directly facilitate neuroplasticity in the central nervous system after passing through the blood brain barrier.

Future studies are warranted to comprehensively investigate the effects of high-intensity interval training on central artery stiffness, neuroplasticity, and cognitive function, and elucidate the associated physiological mechanisms in postmenopausal women, the population most vulnerable to cardiovascular events and cognitive impairment.
